# Good Days, Bad Days: Wind as a Driver of Foraging Success in a Flightless Seabird, the Southern Rockhopper Penguin

**DOI:** 10.1371/journal.pone.0079487

**Published:** 2013-11-13

**Authors:** Nina Dehnhard, Katrin Ludynia, Maud Poisbleau, Laurent Demongin, Petra Quillfeldt

**Affiliations:** 1 Max Planck Institute for Ornithology, Vogelwarte Radolfzell, Radolfzell, Germany; 2 Animal Demography Unit, University of Cape Town, Cape Town, South Africa; 3 Department of Biology - Ethology, University of Antwerp, Wilrijk, Belgium; 4 Behavioural Ecology and Ecophysiology Group, Justus-Liebig University Gießen, Gießen, Germany; Phillip Island Nature Parks, Australia

## Abstract

Due to their restricted foraging range, flightless seabirds are ideal models to study the short-term variability in foraging success in response to environmentally driven food availability. Wind can be a driver of upwelling and food abundance in marine ecosystems such as the Southern Ocean, where wind regime changes due to global warming may have important ecological consequences. Southern rockhopper penguins (*Eudyptes chrysocome*) have undergone a dramatic population decline in the past decades, potentially due to changing environmental conditions. We used a weighbridge system to record daily foraging mass gain (the difference in mean mass of adults leaving the colony in the morning and returning to the colony in the evening) of adult penguins during the chick rearing in two breeding seasons. We related the day-to-day variability in foraging mass gain to ocean wind conditions (wind direction and wind speed) and tested for a relationship between wind speed and sea surface temperature anomaly (SSTA). Foraging mass gain was highly variable among days, but did not differ between breeding seasons, chick rearing stages (guard and crèche) and sexes. It was strongly correlated between males and females, indicating synchronous changes among days. There was a significant interaction of wind direction and wind speed on daily foraging mass gain. Foraging mass gain was highest under moderate to strong winds from westerly directions and under weak winds from easterly directions, while decreasing under stronger easterly winds and storm conditions. Ocean wind speed showed a negative correlation with daily SSTA, suggesting that winds particularly from westerly directions might enhance upwelling and consequently the prey availability in the penguins' foraging areas. Our data emphasize the importance of small-scale, wind-induced patterns in prey availability on foraging success, a widely neglected aspect in seabird foraging studies, which might become more important with increasing changes in climatic variability.

## Introduction

Seabird species are widely used as indicators of environmental conditions and food abundance in the marine environment [1]–[3]. Short-term changes of food availability during the breeding season are often reflected in foraging and provisioning behaviour, such as trip duration and distance travelled, meal sizes and frequency of visits to the nest [4]–[6]. Prey quality and quantity delivered by the parents are critical for nestlings’ growth and their subsequent survival, even after fledging when independent from the parents [7], [8]. Therefore, parents should invest into chick rearing and chick provisioning in order to produce high quality offspring [9]. This energetic investment, however, needs to be traded off with maintenance of their own body reserves [10] to ensure the survival of the adults, especially in long-lived animals like seabirds [11].

Penguins are typical central place foragers during the chick rearing period, and, in comparison to flying seabirds, are more restricted in their foraging range [12]. As such, they are excellent sentinels for local food availability [13]. Adapted to regular provisioning with high-quality food [14], chicks of small-sized penguin species can grow very quickly and fledge (depending on the species and latitude) at an age of about 50 to 98 days [15]. During periods of poor environmental conditions, however, breeding success can drop dramatically up to total breeding failure [16] and adult mortality can increase with serious consequences for population numbers (e.g. [17]).

We studied the daily foraging mass gain (i.e. difference in the mean mass of adults leaving the colony in the mornings and returning in the evening) as a measure of foraging success of southern rockhopper penguins (*Eudyptes chrysocome*) at a breeding site in the Falkland Islands (Islas Malvinas). Southern rockhopper penguins have undergone a dramatic population decline across their sub-antarctic breeding range [18]–[20]. Due to their limited foraging range especially during the chick rearing period (e.g. [21], [22]), they depend on a high productivity in areas adjacent to their breeding sites. Local wind conditions are known to affect the presence of prey species (e.g. [23]) and the ability of seabirds to capture prey [24]. Scientists are now realizing that a change in wind regimes can have important consequences for seabird foraging conditions and consequently their life history [25].

The Falkland Islands are currently located within the west-wind zone, experiencing strong winds from mainly south-westerly to north-westerly directions [26]. The productivity of the marine food web in the western part of the Falkland Islands is positively affected by the western branch of the cold, nutrient rich Falkland Current [27]. Southerly to westerly wind directions coincide with the direction of this current and could contribute to upwelling in this area (see [27] and literature therein), and consequently improve foraging conditions for seabirds. In contrast, it remains unclear how opposing wind directions, particularly from easterly directions affect the foraging conditions for seabirds in this area.

We studied day-to-day variability in adult body mass patterns to compare the daily foraging mass gain of male and female southern rockhopper penguins during different stages of chick rearing and how these changes in body mass are related to wind speed and direction. During guard, only females provision chicks, while males guard the chicks at the nest. During crèche, chicks are provisioned by both parents. As southern rockhopper penguins provision chicks daily [21], [22], short-term differences in prey availability should be reflected in adult foraging mass gain, which in turn could affect chick provisioning. We expect that winds from westerly directions (180−360°) could positively affect upwelling processes and coincide with lower sea surface temperatures. We therefore anticipate higher foraging mass gains under westerly wind directions than under easterly wind directions (0−180°).

## Materials and Methods

The study was conducted in the “Settlement Colony” on New Island, Falkland Islands (51°43’S, 61°17’W), in a colony that held about 7,500 breeding pairs in December 2010.

### Ethics Statement

The marking of penguins with subcutaneous passive integrative transponders (PITs) is a standard field procedure and in the long-term less problematic for penguins than the use of flipper bands [28]. Notably, even much smaller bird species have been studied with PITs, without any noticeable effects [29].

Disturbance during capture and handling of penguins was kept as little as possible by covering the eyes of the penguin with a hood and keeping handling times as short as possible (generally below 20 min). In order to reduce the risk of infection, we carefully sterilized PITs, transponder-injectors and the skin at the injection side, and subsequently glued the skin puncture (VetbondTM, 3M, St. Paul, Minnesota). During the whole period of the study, we never observed any infection at the injection site.

The use of the weighbridge system enabled us to obtain body mass recordings without a constant disturbance due to handling and weighing of penguins in the colony. The position of the weighbridge system at a natural bottleneck path on the way to the colony did not impose the penguins to make a detour or wait any longer than normally.

All work was approved by the Falkland Islands Government (Environmental Planning Office; Research Licenses No: RO09/2006, R16/2007, R05/2009), and we would like to thank the New Island Conservation Trust for permission to work on the island.

### Weighbridge system and body mass analysis

In the course of four consecutive breeding seasons (starting in 2006/07), 753 breeding adult southern rockhopper penguins (380 males, 373 females) were marked with subcutaneous PITs (23 mm length, RFID, Texas Instruments, USA). Each PIT marked bird was measured and weighed, and sex was determined using bill measurements and behaviour [30]. An automated weighbridge system, which reads the PITs and records body mass measurements (see below), has been operating between the landing site and the colony since 2007/08, but only data from 2009/10 and 2010/11 were used in our analysis, as sample sizes for reliable mass data were small in the first breeding seasons. We included the time period between the 11^th^ of December and the 10^th^ of February from these two breeding seasons into this study, which covers the chick rearing period (n = 124 days in total for both seasons, less n = 11 days during which the weighbridge did not work and n = 2 days for which wind data are missing). The weighbridge system records the date, time, PIT-number, and body mass of each crossing PIT bird. In the weighing process, the scale detects up to 6 mass recordings within 0.1 second and logs the average value from these 6 records as one mass recording, unless outliers occurred. A maximum of six mass recordings (from up to 36 individual measurements) were logged per transit of each individual penguin crossing. The balance can detect mass to the nearest 1 g, however body mass recordings are easily impaired by movement of the PIT bird and by potential other penguins on the balance. We carefully scanned the automated weighbridge system files for outliers and only considered crossings with two or more mass recordings. For each penguin crossing, the mean body mass value was calculated, and only included in the analysis if the recorded body mass data per crossing differed by less than 200 g. The remaining outliers were removed. Based on our own body mass measurements when handling birds in the colony (see below, using an electronic spring scale, Kern, Germany, measuring mass to the nearest 10 g) and data published in Williams [12], we assumed the acceptable body mass limits to be 1700–3500 g for females during guard and crèche and 2000–3900 g for males during crèche. This procedure also allowed us to exclude mass recordings obtained by two or more penguins standing on the balance of the weighbridge system.

In order to estimate the general accuracy of these filtered body mass data obtained by the weighbridge system, we compared mass data of manually weighed individuals with those obtained through the weighbridge: In the framework of another concurrent study in the breeding season 2010/11 [22], we manually weighed penguins at their nest-sites (before feeding their chicks during guard and crèche). From 20 of these individuals, we also obtained accurate mass data through the weighbridge system (from the same evening at which the manual weighing took place, and filtered according to the methods described above). The mean body mass of these 20 individuals obtained by manual weighing (2699 g±278 g (SD)), did not differ significantly from the mean body mass obtained through the weighbridge system (2710 g±291 g (SD); paired t-test: t_19_ = 0.43, p = 0.676). Thus, on the population scale, the mass obtained from the weighbridge system is comparable to the mass obtained by manual weighing. During guard and crèche, southern rockhopper penguins on New Island usually leave the colony early in the morning to forage, and start returning later in the morning, with a peak during the afternoon and evening ([21], [31], own observations). Southern rockhopper penguins often crossed through the weighbridge in large groups and then moved rapidly over the weighing balance, so that we rarely obtained accurate mass recordings from the same individual in the morning and in the evening of the same day (even though individual marked birds were recorded in the weighbridge in the morning and in the evening). Therefore, an analysis of the body mass recordings on the individual scale, i.e. determining the daily body mass gain for individual birds, as done in other studies with different penguin species [32]–[34] was not possible. Instead, we worked on the population scale, i.e. we calculated the daily body mass gain from the difference between the mean mass of adults leaving the colony (1:20 to 09:59 hours; peak between 3 and 7 am) and the mean mass of adults returning to the colony (10:00 to 23:40 hours; peak between 4 and 8 pm). Sample sizes (i.e. individuals with accurate mass recordings for leaving or returning) differed among days and between leaving and returning, and were in the range of 15 to 70 birds per set of daily morning (leaving) or evening (return) trips. The daily foraging mass gain therefore reflected the sum of net body mass gain (from self-feeding of the adult) and prey mass that was subsequently fed to the chick.

In the few cases that we have obtained accurate mass recordings from the same individual in the morning and in the evening, we randomly selected one crossing body mass to ensure that each individual was present in the dataset only once on a given day (either in the morning, or in the evening). This procedure guaranteed independent data on a daily basis, while we could not control for individual effects over the course of the breeding season without massively reducing the sample size of mass recordings per day. Considering, however, the relatively minor influence of an individual’s mass recording on the daily foraging mass gain of all birds as calculated in this study, we assumed that this pseudo-replication issue had a negligible effect on our final analysis. We used a paired t-test to check for differences between mean morning (leaving) and mean evening (returning) body mass of adults per day. We further applied an independent t-test to compare the daily foraging mass gain between sexes. Furthermore, we conducted Pearson correlations to test for the concordance in daily foraging mass gain between males and females.

### Wind and SSTA data

Ocean wind and sea surface temperature anomaly (SSTA) data were downloaded from NOAA (http://www.ncdc.noaa.gov/thredds/OceanWinds.html) for the geographic range west of New Island (51–52°S, 61–62.5°W). This area is known to be the main foraging site of southern rockhopper penguins from the study colony during guard and crèche ([21], [22], Ludynia unpublished data). Ocean wind data were based on blending of high-resolution observations from multiple satellites (http://www.ncdc.noaa.gov/oa/rsad/air-sea/seawinds.html#data), and were expressed as U- and V-component of the daily wind speed (in m/s). Wind is thereby expressed as a vector, combining wind speed and wind direction: The V-component describes the wind speed on the North-South axis, with negative values if the wind comes from the North, and the U-component describes the wind speed on the East-West axis, with negative values if the wind comes from the East. Using a trigonometric conversion, these vectors were then transferred into wind direction (in degrees, giving the direction from which the wind was blowing) and daily wind speed (in m/s) along this direction. Wind direction is a circular variable (i.e. wind from 0° equals wind from 360°). In order to test for year differences in wind conditions, we therefore used independent t-tests for the U- and V-component of wind instead of directly using wind speed and wind direction.

SSTA data were calculated as the difference between actual SST and long term average (data from 1971 to 2000) and downloaded from NOAA (http://www.ncdc.noaa.gov/thredds/catalog/oisst/NetCDF/AVHRR/catalog.html). To test for a potential influence of wind speed on daily SSTA through upwelling, we conducted a Pearson correlation test between wind speed and daily SSTA. This test revealed a significant negative correlation (see Results) between the two parameters. To avoid problems with collinearity [35], and as wind should logically drive this relationship, we only included wind speed and wind direction, but not daily SSTA into subsequent statistical analyses.

Data for both breeding seasons were pooled to retain sufficient sample sizes. In order to account for the circularity of wind direction, we conducted a Generalised Additive Model (GAM) in the R package mgcv [36], with daily foraging mass gain (pooled for both sexes) as dependent variable. As explanatory variables, we included breeding season (2009/10 or 2010/11) and chick rearing stage (guard or crèche) as fixed factors, wind speed (in m/s, as numerical) and wind direction (in degrees; the circularity was accounted for by a circular smoother) and the two-way interaction between wind speed and wind direction (again, the circularity of the wind direction was accounted for by a circular smoother).

Statistical analyses were run in R 3.0.1. [37]. Means are given with standard deviation throughout the manuscript.

## Results

**Figure 1:**
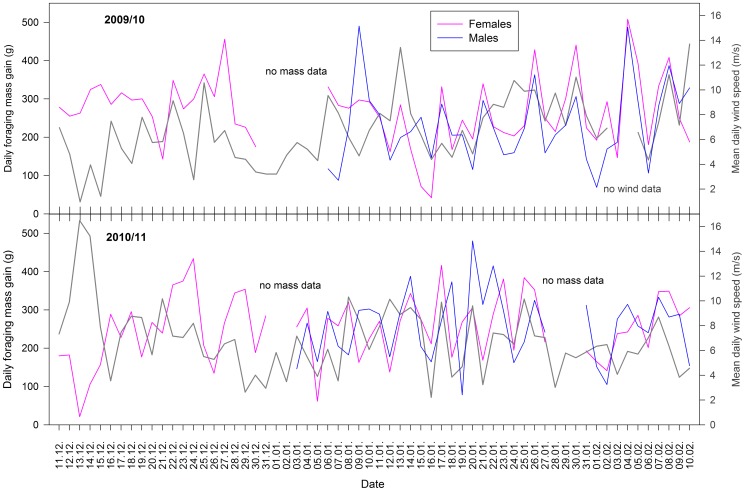
Daily foraging mass gain with wind speed across time. Daily foraging mass gain (in g) of females (in pink) and males (in blue) against wind speed (in dark grey) in the breeding seasons 2009/10 (top) and 2010/11 (below). Daily foraging mass gain was calculated as the difference between mean mass of adults leaving the colony in the mornings and mean mass of adults returning to the colony in the evenings.

Males and females that crossed the weighbridge system when returning from foraging were significantly (between 243 and287 g) heavier than when leaving (paired t-test: t_184_ = 38.14, p<0.001; Table 1). During the crèche stages, daily foraging mass gain did not differ significantly between sexes (t-test: t_142_ = 0.283 and p = 0.777), but was highly correlated between males and females (r = 0.49, p<0.001, n = 72 days), indicating synchronous changes among days within males and females (Fig. 1). We therefore pooled daily foraging mass gain for both sexes in the subsequent analyses.

**Table 1:** Foraging mass gain for sexes and chick rearing stages.

	Females		Males	
	Mean ± SD	Range	Mean ± SD	Range
**Guard 2009/10**	287±68	143–456		
**Crèche 2009/10**	260±99	42–508	251±108	75–353
**Guard 2010/11**	243±101	21–434		
**Crèche 2010/11**	258±79	62–416	257±88	78–480

Foraging mass gain in g (i.e. difference between evening and morning body mass; body mass of individuals was pooled for both mornings and evenings) of adult southern rockhopper penguins crossing the weighbridge system at New Island, Falkland Islands. Data were obtained through the weighbridge system from n = 316 individual females and n = 276 individual males in 2009/10 and n = 330 individual females and n = 301 individual males in 2010/11.

**Figure 2:**
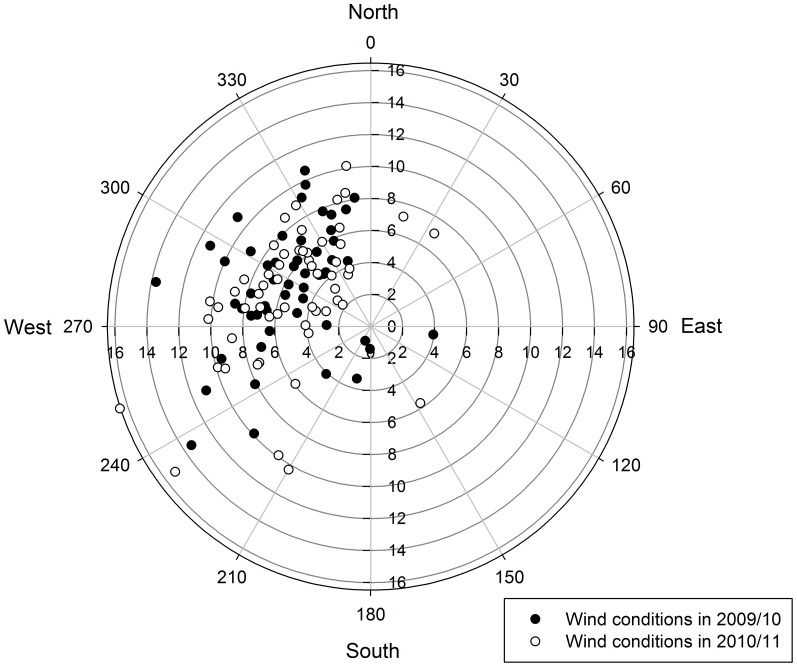
Wind conditions during the breeding season 2009/10 and 2010/11 (n = 111 days). The position of scatter plot points within the windrose represent the direction from which the wind was blowing, while the distance from the origin represents the wind speed (in m/s).

Mean daily ocean wind speed showed a high variability among days (Fig. 1), and was dominated by south-westerly to north-westerly wind directions in both years (Fig. 2). Wind conditions between the two years did not differ significantly (t-tests for the U- and V-component of wind; both t_109_ ≥ |0.33|, P ≥ 0.509). Mean daily wind speed was negatively correlated with daily SSTA (r = –0.33, p<0.001, n = 111).

**Figure 3:**
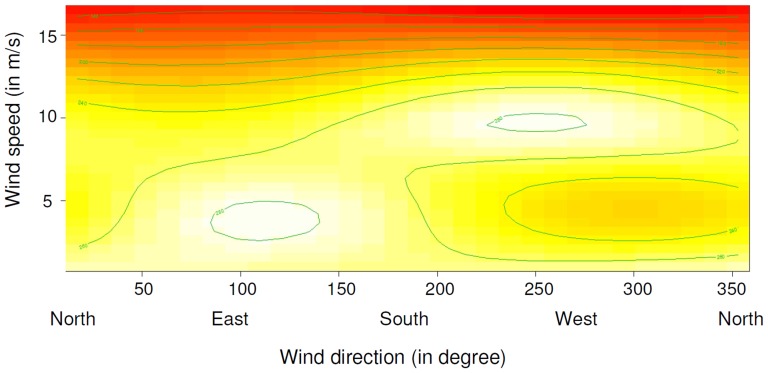
Relationship between daily wind speed and daily wind direction on the daily foraging mass gain of southern rockhopper penguins. The graphical output of the GAM (see Table 2 for details) shows foraging mass gain as a colour scale ranging from high foraging success in white to low foraging success in red, depending on wind speed (y-axis) and wind direction (x-axis).

Daily foraging mass gain showed a high variation across time within both breeding seasons (Table 1, Fig. 1), but did not differ significantly between breeding seasons and chick rearing stages (Table 2). The interaction between wind direction and wind speed had a significant effect on the daily foraging mass gain (Table 2). Under north-easterly to south-easterly wind directions (0−180°), the foraging mass gain of southern rockhopper penguins was highest under low wind speeds (Fig. 3). With increasing wind speeds from the easterly range, foraging mass gain decreased. For the westerly wind range (180−360°), foraging mass gain increased from weak to moderate wind speeds. Under storm conditions (mean daily wind speed of ≥ 13 m/s), foraging mass gain was lowest throughout all wind directions.

**Table 2:** Results for the GAM with daily foraging mass gain as dependent variable.

Explanatory variables	Df	F	P
Breeding season	1	0.726	0.396
Chick rearing stage	1	0.865	0.355
Wind speed	**1**	**0.692**	**0.010**
Wind direction	1	<0.001	0.597
Wind speed*wind direction	**2.2**	**0.682**	**0.001**

Breeding season (2009/10 or 2010/11) and chick rearing stage (guard or crèche) were included as fixed factors, wind speed (in m/s) and wind direction (in degree, circularity was accounted for by a circular smoother) were included as continuous variables. In addition, we included the interaction between wind speed and wind direction (again accounting for the circularity with a smoother). n = 111 days, the model explained 15.8% of the deviance. Significant results are marked in bold.

## Discussion

In the present study, daily foraging mass gain as an indicator of foraging success of adult southern rockhopper penguins was highly variable over time, while showing no significant differences between breeding seasons, chick rearing stages and sexes. The high degree of day-to-day variation in foraging mass gain suggests that local foraging conditions for penguins were variable among days. This is further supported by the fact that daily foraging mass gain was highly correlated between males and females, indicating that both sexes experienced similar foraging conditions. In fact, GPS tracking has revealed that males and females use the same foraging areas in the west of New Island during crèche (Ludynia unpublished observations).

Our data indicate, however, that day-to-day variation in foraging mass gain of southern rockhopper penguins is linked to the local wind conditions, which in turn affect SSTA. We found that the penguins’ daily foraging mass gain was significantly affected by a combination of wind speed and wind direction, as indicated by the significant interaction of these two variables. Foraging conditions were better under moderate to strong winds from westerly directions and low winds from easterly directions. Under storm conditions, foraging mass gain declined throughout all wind directions.

The effect of wind patterns should not significantly affect travel time of flightless penguins as could be expected for flying seabird species [25], [38]. Even if ocean wave action and increased counter-currents due to certain wind conditions could affect the swim speed of the penguins, an effect on the travel time on the outcomes of this study should be minimal for several reasons: Firstly, due to friction loss, the effect of wind on wave action and counter-currents and finally on the swim speed of the penguins should be smaller than the comparable direct effect of wind on a flying bird. Secondly, the distances that rockhopper penguins travel on their daily foraging trips during the chick rearing period are short (about 30 to 60 km within about 12 hours [22]) and travel times should therefore hardly be affected by opposing wind conditions, particularly when considering the enormous distances that these birds can travel in short times (e.g. 500−1600 km within 10−19 days during the incubation foraging trip [39]). Thirdly, foraging grounds of southern rockhopper penguins breeding in our study colony are located in the west of the breeding colony. If wind conditions would affect the foraging mass gain through travel time, one would expect a negative effect of westerly winds ( =  headwinds) on the daily foraging mass gain, which was only found during storm conditions.

Instead, the penguins' foraging success should rather be affected by oceanographic conditions and prey availability. Moderate to strong winds are known to cause mixing of the water column and local upwelling in the open ocean [40]. In coastal areas, upwelling is determined by the course of the coastline as well as currents in relation to wind directions [40]. Upwelling of cold, nutrient rich water from deeper strata leads to an increased primary productivity, i.e. growth of phytoplankton in the photic zone [40]. This will in turn attract higher consumer levels [41]. Moreover, changes in the stratification of the water column (e.g. through wind) can also directly affect the distribution of zooplankton [41], as well as the abilities of diving seabirds to capture prey [42]. Zooplankton will attract larger prey species and thus seabirds [24], whose feeding rates might also depend directly on wind directions [43] and the presence of oceanographic fronts [44], [45].

In the Falkland Islands, southerly to westerly wind directions coincide with the direction of cold, nutrient rich waters from the Falkland Current and might contribute to upwelling in this area (see [27] and literature therein). In fact, we found a negative correlation between mean daily wind speed and daily SSTA, suggesting that stronger winds (which in our dataset mostly came from south-westerly to north-westerly directions; see Fig. 2) lead to enhanced upwelling of cold, nutrient rich water. This agrees with our finding of increased foraging mass gain under moderate to strong westerly winds (Fig. 3). On the contrary, for the easterly wind directions (opposing the Falkland Current and upwelling), the optimum foraging mass gain coincided wind speeds of 5 m/s and less. Such weak winds (equivalent to 3 Beaufort and less) do not yet cause the formation of waves [46], and likely have no influence on upwelling processes. Our findings further suggest that moderate to strong wind speeds from easterly wind directions lead to a reduction in the daily foraging mass gain, potentially as these winds reduce upwelling.

In addition, our data illustrate that under storm conditions southern rockhopper penguins forage less successfully. This might be caused by an effect of strong waves on the foraging ability of the penguins. Furthermore, strong waves under storm conditions hinder the save entry and exit at landing sites and pose a high risk of injury on rockhopper penguins trying to come ashore.

### Conclusions on foraging behaviour and chick provisioning

Southern rockhopper penguins are opportunistic feeders that take a mixture of krill, squid and fish, but proportions of prey items vary strongly over time and between locations [47]–[50]. Although energetic requirements of adults and juveniles can influence the prey choice [51], this broad food spectrum might also be the consequence of a high variability in prey type availability or abundance [52].

For the daily foraging mass gain recorded in this study, we did not quantify the exact proportions of how much food was used for self-feeding of the adult birds and how much prey was fed to the chicks. The daily foraging mass gain that we recorded was on average higher than the mean stomach content wet mass found in southern rockhopper penguins breeding on the Falkland Islands (mean stomach content wet mass of 79 to 221 g, depending on study site [49], [53]) and on Staten Island, Argentina (mean stomach content wet mass of 101 and 106 g, depending on breeding stage [54]). This suggests that a considerable amount of the captured prey was not actually delivered to the chicks but digested by adults, potentially still during the foraging trip ([55] and literature therein).

The food demand of chicks increases with age and size and also depends on the food quality [14]. The average food intake of captive African penguin chicks (*Spheniscus demersus*) was 20–30% of their own body mass (see [14]). Applied to our study, southern rockhopper penguin chicks might take up to 500–750 g of food per day at the peak of their growth curve when they are about 50 to 60 days old (body mass of about 2500 g; see [56] for a chick growth curve of southern rockhopper penguins). If this is the case, the chicks’ food demand could exceed the daily foraging mass gain of both parents (compare with Table 1). Yet, this high food demand is limited to a short time period (during crèche) during which both parents contribute to chick rearing. Interestingly, despite the increasing food demand of chicks with progressing breeding season, we did not observe an increase in the daily foraging mass gain with the progress of the breeding season. This could be linked to self-allocation of prey by adults to regain body mass reserves after fasting during incubation (females) and guard (males) [39].

### Implications for population trends

The Falkland Islands are currently located in the area of the southern ocean west wind drift [57]. Climate change scenarios, however, predict an increase of wind speeds and a southward shift of this wind zone [57], which already affects life history traits of wandering albatrosses (*Diomedea exulans*) breeding on the Crozet Islands [25]. It seems likely that a poleward shift of the west wind drift will reduce the number of days with westerly winds on the Falkland Islands in the future. For southern rockhopper penguins breeding on the Falkland Islands, this would imply fewer days with favourable foraging conditions, as indicated by our data. Moreover, other seabird species breeding on the Falkland Islands, including black-browed albatrosses (*Thalassarche melanophris*) and several species of petrels, which are depending on high wind speeds [58], are likely to be negatively affected by the changing wind conditions. On a more global scale, one can expect that the shift of wind regimes due to global warming will affect seabird species all over the world, including southern rockhopper penguins at different breeding sites.

The population of southern rockhopper penguins is listed as vulnerable [19]. The breeding population on the Falkland Islands has declined from about 1.5 million breeding pairs [18] in the 1930s to about 210,000 breeding pairs in November 2005 [59]. Even though the population has meanwhile increased to about 319,000 breeding pairs in November 2010 [59], the exact reasons for the original decline still have to be identified. Increasing SST due to global warming might play an important role [19], [60]. As such, this study may add important information about an additional global change related threat to rockhopper penguins. The poleward shift of the west wind drift might deteriorate foraging conditions close to breeding colonies of southern rockhopper penguins in the future. Other seabird species, including the closely related macaroni penguins (*Eudyptes chrysolophus*), have reacted to changes in local food availability by shifting to another prey type or extending their foraging trips [61]. Despite these adaptations, reduced local food availability resulted in reduced breeding success and/or fledglings were significantly lighter than under normal food conditions [61]. Environmental changes leading to reduced food availability close to penguin colonies are therefore assumed to have negative consequences for future population trends (e.g. see [62] for king penguins *Aptenodytes patagonicus*), and this can also be assumed for southern rockhopper penguins.
